# γδ T Cells Mediate Protective Immunity Following Vaccination with an Insect-Based Chikungunya Fever Vaccine in Mice

**DOI:** 10.3390/pathogens14090863

**Published:** 2025-08-30

**Authors:** Leslie Rodriguez, Awadalkareem Adam, Huanle Luo, Samantha R. Osman, Kenneth Plante, Shannan L. Rossi, Scott C. Weaver, Tian Wang

**Affiliations:** 1 Department of Microbiology & Immunology, University of Texas Medical Branch, Galveston, TX 77555, USA; lesrodri@utmb.edu (L.R.); awadam@utmb.edu (A.A.); luohle@mail.sysu.edu.cn (H.L.); samanthaosman@alumni.rice.edu (S.R.O.); ksplante@utmb.edu (K.P.); sweaver@utmb.edu (S.C.W.); 2 Sealy Institute for Vaccine Sciences, University of Texas Medical Branch, Galveston, TX 77555, USA; slrossi@utmb.edu; 3 World Reference Center for Emerging Viruses and Arboviruses, University of Texas Medical Branch, Galveston, TX 77555, USA; 4 Department of Pathology, University of Texas Medical Branch, Galveston, TX 77555, USA; 5 Institute for Human Infections and Immunity, University of Texas Medical Branch, Galveston, TX 77555, USA

**Keywords:** chikungunya virus, vaccines, γδ T cells, protective immunity, T cells, antibody

## Abstract

Eilat (EILV)/chikungunya virus (CHIKV) is a chimeric virus that contains the nonstructural proteins and cis-acting sequences of EILV and the structural proteins of CHIKV. EILV/CHIKV vaccination is known to protect with a single dose against wild-type (WT) CHIKV challenge in mice and non-human primates. The underlying immune mechanism of the vaccine-induced host protection remains unknown. γδ T cells react to WT CHIKV infection by controlling the virus-induced tissue inflammation and damage. Here, we found that γδ T cells contribute to EILV/CHIKV-induced host protection against WT CHIKV infection. TCRδ^−/−^ mice, which are deficient of γδ T cells, had impaired CHIKV-specific CD8^+^ T cell responses, antibody production and memory B cell responses following vaccination. Both antibody and CD8^+^ T cells of EILV/CHIKV-vaccinated mice were required for protection type I interferon receptor deficient mice from lethal WT CHIKV infection. Moreover, γδ T cells expanded quickly in response to EILV/CHIKV vaccination. TCRδ^−/−^ mice, had lower levels of innate immune cytokines and impaired activation of antigen presenting cell (APCs). Overall, γδ T cells contribute to EILV/CHIKV-induced host protection by promoting APC maturation, T cell priming and the induction of humoral immune responses upon EILV/CHIKV vaccination.

## 1. Introduction

Chikungunya virus (CHIKV; species *Alphavirus chikungunya*), a re-emerging mosquito-borne, positive-sense RNA virus, belongs to the genus *Alphavirus* of the family *Togaviridae*. CHIKV has a 5′ capped RNA genome of 11.8 kb in length that encodes four nonstructural proteins (nsP1–4) and six structural proteins, including the capsid protein (C), envelope (E) glycoproteins (E3, E2, and E1), 6K viroporin channel, and transframe proteins (TF) [1,2,3,4,5]. The virus was initially isolated in 1952 in what is now Tanzania and has caused epidemics in Africa, Asia, Europe, and the Americas, with millions of human cases. The virus induces acute and chronic human infections with main clinical symptoms including the flu-like chikungunya fever (CHIKF) and chronic joint pain [6,7]. An effective vaccine is needed to prevent or reduce CHIKV-associated chronic morbidity. Multiple platforms have been used for CHIKV vaccine development, including formalin-inactivated, virus-like particles (VLP), recombinant subunit, DNA, virus-vector-based, live-attenuated, and chimeric *alphavirus*-based vaccines [8,9,10,11,12,13,14,15,16,17]. Recently, two vaccines, Ixchiq [10], a live-attenuated CHIKV vaccine and VIMKUNYA [18], a virus-like particle were both approved for human use by the U.S. Food and Drug Administration. Despite this achievement, there is age restriction for application of these vaccines due to safety limits on the age groups included in the clinical trials. Furthermore, limited data are available on the long-term effectiveness and safety of these vaccines. Thus, additional CHIKV vaccination strategies are needed.

Eilat virus (EILV) is an insect-specific alphavirus that is defective for replication in vertebrate cells [19]. EILV/CHIKV, a chimeric virus, was previously constructed to include the EILV 5′ and 3′ untranslated regions (UTRs), subgenomic promoter, and the NS proteins (nsP1, nsP2, nsP3 and nsP4) of EILV and the structural proteins of CHIKV (E1, 6K/TF, E2, E3 and capsid). The chimeric virus replicates to very high titers in mosquito cells but cannot replicate in vertebrate cells [20,21]. A single dose of EILV/CHIKV induces cellular and humoral immunity and protects mice from CHIKV-induced disease up to 9.5 months post vaccination. Furthermore, EILV/CHIKV completely protects cynomolgus macaques from CHIKF up to one year post vaccination [22]. Despite these successful efforts, the underlying mechanisms of immune induction by the EILV chimera remain unclear.

γδ T cells comprise a minority of the CD3^+^ T cells in lymphoid tissue and blood in humans and rodents [14]. Unlike αβ T cells, they lack MHC restriction and have the capacity to respond to antigens without a requirement for conventional antigen processing [15]. In response to microbial infection, γδ T cells can rapidly produce cytokines, such as IFN-γ and TNF-α [7,8]. These unique features suggest that γδ T cells play a role in innate immunity during microbial infection. They are also known to form a link between innate and adaptive immune responses [9,10,11,12]. γδ T cells are also reactive following wild-type CHIKV infection [23]. Here, we investigated the role of these cells in mediating protective immunity following EILV/CHIKV vaccination. Our results suggest that γδ T cell-mediated CD8^+^ T cell and humoral immune responses contribute to EILV/CHIKV-induced host protection against WT CHIKV infection.

## 2. Materials and Methods

### 2.1. Mice

Wild-type (WT) C57BL/6 (B6) mice, B6 mice deficient in γδ T cells (TCRδ^−/−^) and B6 mice deficient in the IFN-α/β receptor (AB6) were bred and maintained at the University of Texas Medical Branch (UTMB) animal facility. Mice were inoculated intraperitoneally (i.p.) with 10^8^ PFU EILV/CHIKV or PBS (mock). In some experiments, vaccinated mice were challenged i.p. with the WT-CHIKV La Réunion (LR) strain. All experiments were performed with 6- to 8-week-old animals. Groups were age-matched for each vaccination or infection. All animal experiments were approved by the Animal Care and Use Committee at UTMB.

### 2.2. Virus

EILV/CHIKV was grown in C7/10 cells and purified as described in our prior studies [24]. The WT CHIKV LR strain stock was kindly provided by Dr. Trevor Brasel at UTMB, which was passaged once and titrated in Vero cells.

### 2.3. Intracellular Cytokine Staining (ICS) and Flow Cytometry Analysis

Splenocytes from vaccinated mice or controls were stimulated with the CD8^+^ T cell-restricted CHIKV E1 peptide (HSMTNAVTI) [17] for 5 h at 37 °C. Golgi-plug (BD Biosciences, Franklin Lakes, NJ, USA) was added at the beginning of stimulation. Cells were harvested, stained with Abs for CD8 (clone 53-6.7, # 11-0081-81, Thermo Fisher), and CD3e (clone 145-2C11, # 12-0031-81, Thermo Fisher), and fixed in 2% paraformaldehyde (PFA) and permeabilized with 0.5% saponin before adding PE-conjugated anti-IFNγ (clone XMG1.2, # 17-7311-82) or mouse IgG1. In some experiments, splenocytes of vaccinated WT and TCRδ^−/−^ mice were isolated and stained for TCRγ/δ, CD3e, CD8α, CD11c, CD80, CD86, F4/80 (ThermoFisher, San Diego, CA, USA). After staining, the cells were fixed in 2% PFA. All samples were acquired and analyzed using a C6 flow cytometer (BD Biosciences). Dead cells were excluded on the basis of forward and side light scatter.

### 2.4. Plaque Assay

Vero cells were seeded in 12-well plates at 0.2 × 10^6^ cells per well overnight at 37 °C. Cells were infected with 10-fold serial dilutions of sera collected from infected mice at 37 °C with 5% CO_2_ for 1 h. After the incubation, 0.4% agarose overlay medium was added to the infected cells. The plates were incubated at 37 °C with 5% CO_2_ for 48 h. Plates were fixed with 10% formalin for 30 min. Cells were stained with 0.25% crystal violet for 5 min and then rinsed with H_2_O. Plaques were counted and calculated as PFU/mL. Viral titers were calculated by using the following formula: PFU/mL = (average number of plaques/volume [mL] of viral dilution) × dilution factor.

### 2.5. ELISA

Sera from vaccinated mice were collected at indicated time points. The plates were coated with EILV/CHIKV (5  × 10^4^ PFU per well) or 100 ng/well recombinant CHIKV E2 protein (SinoBiological, Houston, TX, USA) overnight at 4 °C, as described previously [22], washed twice with PBS containing 0.05% Tween-20 (PBS-T), and blocked with 8% FBS for 2.5 h. Sera diluted 1:100 in blocking buffer was added for 1 h followed by incubation with goat anti-mouse IgG (# A3562, Sigma Aldrich, St. Louis, MO, USA) coupled to alkaline phosphatase (1:1000 dilution) for 1 h. Color was developed with *p*-nitrophenyl phosphate (Sigma-Aldrich) and the intensity was read at an absorbance of 405 nm.

### 2.6. Plaque Reduction Neutralization (PRNT) Assay

Fifty percent plaque-reduction neutralization tests (PRNT_50_) were performed on Vero cells. Briefly, mouse sera were heat-inactivated at 56 °C for 30 min and then serially diluted (2-fold dilutions) in 2% FBS MEM media. CHIKV LR strain stock was diluted to 800 PFU/mL. Then 150 µL of the diluted sera was mixed with 150 µL of virus stock and incubated at 37 °C for 60 min. Afterwards, 100 μL of each sera/virus mixture was added to Vero cells and incubated for 1 h at 37 °C, with rocking every 15 min. An overlay of MEM/0.4% agarose was added to each well and incubated at 37 °C with 5% CO_2_ until plaques appeared. Plates were fixed with 10% formaldehyde and stained with 0.25% crystal violet. The plaques were counted and the PRNT_50_ titers were calculated as the highest dilution of serum that inhibited 50% of plaques compared to unneutralized samples.

### 2.7. B Cell ELISPOT Assay

ELISpot assays were performed as previously described [25]. Briefly, splenocytes were seeded in 48-well plates and were stimulated with 1 µg/mL R848 and 10 ng/mL recombinant human IL-2 (Mabtech Inc., Cincinnati, OH, USA). Millipore ELISPOT plates (Millipore Ltd., Darmstadt, Germany) were coated with a peptide pool of the CHIKV structural proteins (core, E3, E2, and E1), including 15 amino acid (aa) peptides (15 µg/mL, Sigma, St. Louis, MO, USA) with overlapping 12 aa or with EILV/CHIKV (1 × 10^8^ PFU/well). Cells were harvested and added in duplicates to assess CHIKV-specific IgG antibody-secreting cells (ASC)s. The plates were incubated overnight at 37 °C, followed by biotin conjugated anti-mouse IgG (Mabtech Inc.) for 2 h at room temperature and then streptavidin–ALP for 1 h. Plates were developed with BCIP/NBT-Plus substrate until distinct spots emerged, washed and scanned using an ImmunoSpot 4.0 analyzer; the spots were counted with ImmunoSpot software (Cellular Technology Ltd., Cleveland, OH, USA).

### 2.8. Quantitative PCR (Q-PCR) for Cytokine Analysis

Blood cells were re-suspended in Trizol (Invitrogen, Carlsbad, CA, USA) for RNA extraction. Complementary (c) DNA was synthesized by using a qScript cDNA synthesis kit (Bio-Rad, Hercules, CA, USA). The sequence of the primer sets for cytokines and PCR reaction conditions were described previously [24,26,27,28]. The PCR assay was performed in the CFX96 real-time PCR system (Bio-Rad). Gene expression was calculated using the formula 2^ ^−[C^t^(target gene)−C^t^(*β-actin*)]^ as described before [29].

### 2.9. Adoptive Transfer of CD8^+^ T Cells

As described previously [30,31], single-cell suspensions of CD8^+^ T cells were prepared from spleens of EILV/CHIKV-vaccinated WT or TCRδ^−/−^ mice by negative selection using magnetic beads (Miltenyi Biotec, Bergisch Gladbach, Germany). A total of 7 × 10^6^ cells were i.p. injected into naive 6-week-old AB6 mice 24 h before infection with 80 PFU of WT CHIKV LR. After challenge, the infected mice were monitored daily for morbidity and mortality.

### 2.10. Passive Immunization and Adoptive Transfer

Six-week-old AB6 mice were transferred i.p. with pooled sera from mock or vaccinated WT or TCRδ^−/−^ mice diluted 1:3 in PBSG 24 h before challenge i.p. with 1 × 10^3^ PFU WT CHIKV LR strain. Mice were anesthetized by isoflurane inhalation during adoptive transfer and infection. In some experiments, 3.3 × 10^6^ of purified CD8^+^ T cells from vaccinated mice were transferred i.p. together with pooled immune sera diluted 1:3 in PBSG. Infected animals were monitored daily for clinical signs and weight loss and were euthanized by CO_2_ inhalation if they became moribund. All surviving animals were euthanized via CO_2_ inhalation 3 weeks post infection.

### 2.11. Statistical Analysis

Survival curve comparison was performed using GraphPad Prism software 10.4.2, which uses the log-rank test. Values for viral load, cytokine production, memory B cell frequency, antibody titers, and T cell response experiments were compared using Prism software statistical analysis and were presented as means ± SEM. *p* values of these experiments were calculated with a non-paired Student’s *t* test.

## 3. Results

### 3.1. γδ T Cells Contribute to EILV/CHIKV-Mediated Host Protection upon WT CHIKV Infection in Mice

**Figure 1:**
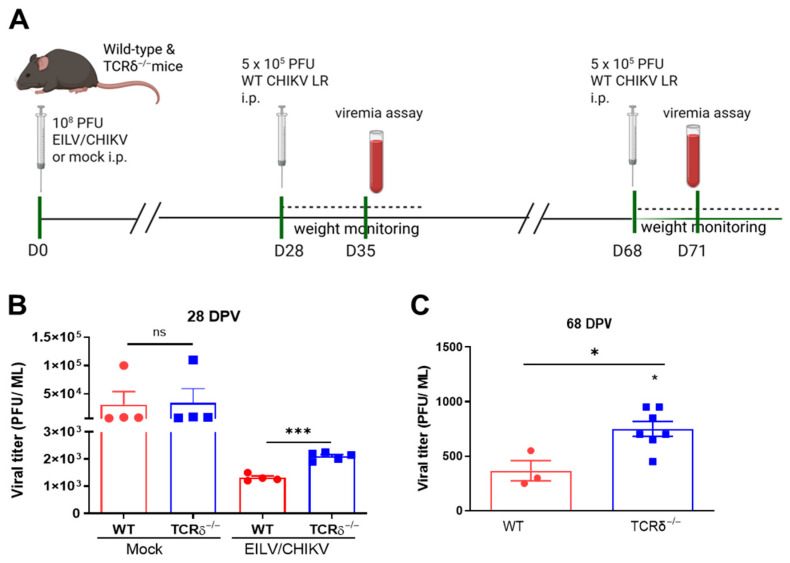
Role of γδ T cells in EILV/CHIKV-induced host protection against CHIKV infection. WT and TCRδ^−/−^ mice were vaccinated with 10^8^ PFU EILV/CHIKV or PBS (mock). At 28 and 68 days post vaccination (DPV), mice were challenged i.p. with 5 × 10^5^ PFU of WT CHIKV LR strain. Mice were monitored daily for morbidity and weight changes. (**A**). Study design (Created in BioRender. Wang, T. (2025) https://BioRender.com/8g9wbga (accessed on 15 August 2025)). (**B**,**C**)**.** Viremia at day 7 (**B**) and day 3 (**C**) post CHIKV infection were determined by plaque assay in mice challenged at 28 and 68 DPV. *** *p* < 0.001 or * p < 0.05 compared to WT group.

Vaccination with EILV/CHIKV has been shown to protect the host from wild-type (WT) CHIKV challenge [21,22]. To understand the role of γδ T cells in EILV/CHIKV-induced host protection, we vaccinated WT C57BL/6 and TCRδ^−/−^ mice, which are deficient of γδ T cells, with a single 10^8^ PFU dose of EILV/CHIKV or PBS (mock). At 28 or 68 days post vaccination (DPV), all animals were challenged with 5 × 10^5^ PFU of the WT CHIKV LR strain (Figure 1A). No difference in viremia was noted between the mock-vaccinated WT and TCRδ^−/−^ mice following WT CHIKV infection. EILV/CHIKV-vaccinated TCRδ^−/−^ mice infected with WT CHIKV at 28 DPV showed significantly higher viremia at day 7 post-CHIKV infection and had significantly more weight loss starting day 2 post infection than those of WT mice (Figure 1B and Figure S1A). Moreover, EILV/CHIKV-vaccinated TCRδ^−/−^ mice infected at 68 DPV also showed increased viremia at day 3 post-infection and a trend of more weight loss compared to EILV/CHIKV-vaccinated WT mice (Figure 1C and Figure S1B). All mice survived 3 weeks post WT CHIKV infection. These results suggest that γδ T cells are required for EILV/CHIKV.

### 3.2. TCRδ^−/−^ Mice Had Impaired CHIKV-Specific CD8^+^ T Cell Responses Following EILV/CHIKV Vaccination and/or Wild-Type CHIKV Challenge

**Figure 2:**
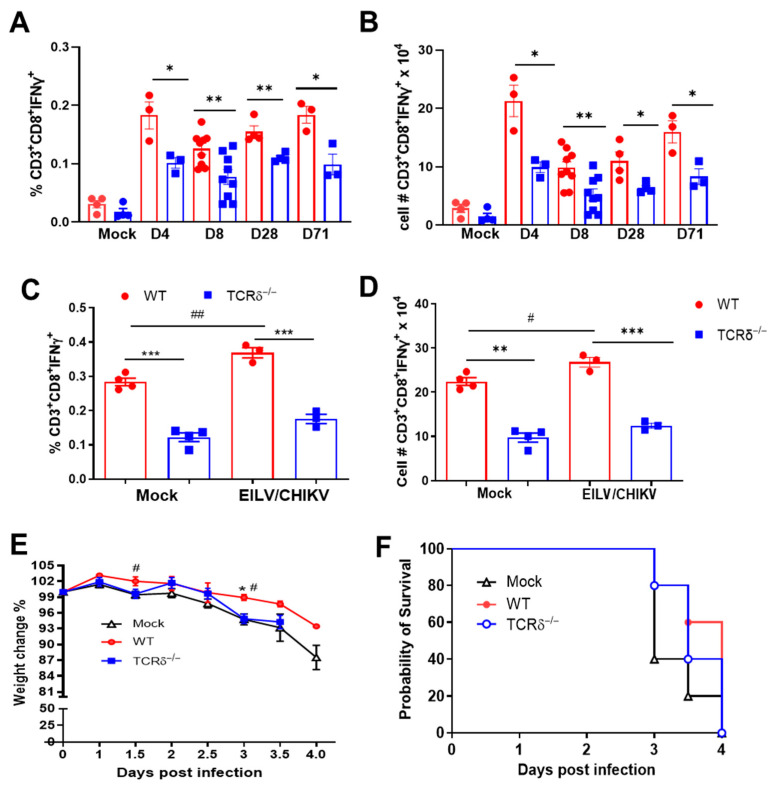
CD8^+^ T cell responses in WT and TCRδ^−/−^ mice following EILV/CHIKV vaccination and WT CHIKV challenge. WT and TCRδ^−/−^ mice were vaccinated i.p. with 10^8^ PFU EILV/CHIKV, or PBS (mock). At 28 DPV, some mice were challenged with 5 × 10^5^ PFU WT CHIKV LR. (**A**–**D**). At 4, 8, 28 and 71 DPV (**A**,**B**) or day 7 post WT CHIKV challenge (**C**,**D**), splenocytes were stimulated with CHIKV E1 peptide for 5 h, and stained for IFN-γ, CD3, and CD8. Total T cells were gated. Percent positive and total number of IFN-γ^+^ CD8^+^ T cells per spleen are shown. n = 3 to 9. (**E**,**F**). At 28 DPV, CD8^+^ T cells of vaccinated WT and TCRδ^−/−^ mice were isolated and transferred to AB6 mice 24 h before infection i.p. with 80 PFU WT CHIKV LR strain. Mice were monitored daily for weight changes and survival rate (n = 5 to 10). Weight changes are indicated by percentage using the weight on the day of infection as 100%. *** *p* < 0.001, ** *p* < 0.01, or * *p* < 0.05 compared to WT group. ## *p* < 0.01, or # *p* < 0.05 compared to mock group.

CHIKV-specific CD8^+^ T cell immunity contributes to viral clearance and protection of the host from footpad swelling and reduced inflammation [32,33]. To determine if γδ T cells mediate CD8^+^ T cell responses following vaccination, mice were vaccinated with 10^8^ PFU of EILV/CHIKV or PBS (mock). At 4, 8, 28, and 71 DPV, spleens were collected to measure CHIKV-specific CD8 T cell responses. Both groups of mice showed strong CHIKV-specific CD8^+^ T cell responses post vaccination compared to the mock-group. EILV/CHIKV-vaccinated TCRδ^−/−^ mice exhibited a significantly lower CD8^+^ T cell response than the vaccinated WT mice at all time points (Figure 2A,B). At 28 DPV, mice were challenged with WT CHIKV. At day 7 post challenge, TCRδ^−/−^ mice exhibited reduced CHIKV-specific CD8^+^ T cell responses compared to those of WT mice (Figure 2C,D). To determine the protective efficacy of γδ T cell-mediated CD8^+^ T cell response against CHIKV infection, we purified CD8^+^ T cells from WT and TCRδ^−/−^ mice 28 DPV (Figure S2A) and adoptively transferred them into 6-week-old IFN-α/β receptor (AB6) mice one day before challenge with 80 PFU of the CHIKV WT LR strain. Mice transferred with WT CD8^+^ T cells showed reduced weight loss at days 1.5 and 3 compared to the mock group. In addition, mice transferred with WT CD8^+^ T cells had reduced weigh loss compared to the TCRδ^−/−^ group at day 3 (Figure 2E). Neither group showed protection from WT CHIKV-induced mortality and no differences were noted between the WT and TCRδ^−/−^ groups (Figure 2F). Tregs expansion is also known to control CHIKV-mediated immunopathology [34]. We assessed the expansion of CD4^+^ Tregs in EILV/CHIKV-vaccinated wild-type and TCRδ^−/−^ mice. At 4, 8 and 28 DPV, no expansion of Tregs was noted. No differences in Tregs expansion were noted between the two vaccinated groups (Figure S2B). These results together suggest that γδ T cells are not involved in Treg expansion after EILV/CHIKV vaccination. Furthermore, γδ T cell-mediated CD8^+^ T cell responses partially contribute to host protection against WT CHIKV infection.

### 3.3. TCRδ^−/−^ Mice Displayed Impaired CHIKV-Specific Antibody and Memory B Cell Responses Following EILV/CHIKV Vaccination

**Figure 3:**
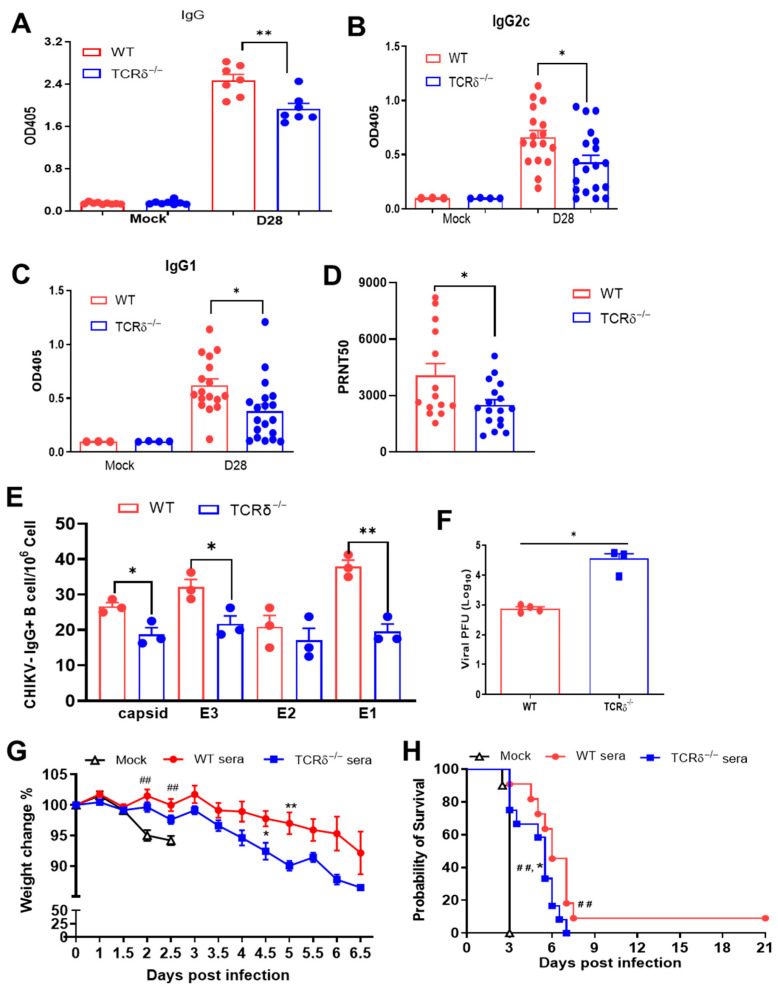
Antibody and memory B cell responses in WT and TCRδ^−/−^ mice following EILV/CHIKV vaccination. Mice were vaccinated i.p. with 10^8^ PFU EILV/CHIKV, or PBS (mock). (**A**–**C**). At 28 DPV, EILV/CHIKV-specific IgG, CHIKV E2-specific IgG2c and IgG1 antibodies in sera were detected as O.D. values by ELISA. (**D**). PRNT_50_ against the CHIKV LR strain were determined by plaque reduction neutralization test. n = 7 to 13. (**E**). At 71 DPV, CHIKV-specific MBC responses were determined by ELISPOT analysis. Frequencies of CHIKV antibody-secreting cells per 10^6^ cells were shown. n = 3. (**F**–**H**). At 28 DPV, pooled sera of EILV/CHIKV-vaccinated WT and TCRδ^−/−^ mice were transferred to AB6 mice 24 h before infection i.p. with 1000 PFU WT CHIKV LR strain. (**F**). Day 3 viremia were determined by plaque assay. (**G**,**H**). Mice were monitored daily for weight changes and survival rate (n = 10 to 12). Weight changes are indicated by percentage using the weight on the day of infection as 100%. ** *p* < 0.01, or * *p* < 0.05 compared to WT group. ## *p* < 0.01 compared to mock group.

**Figure 4:**
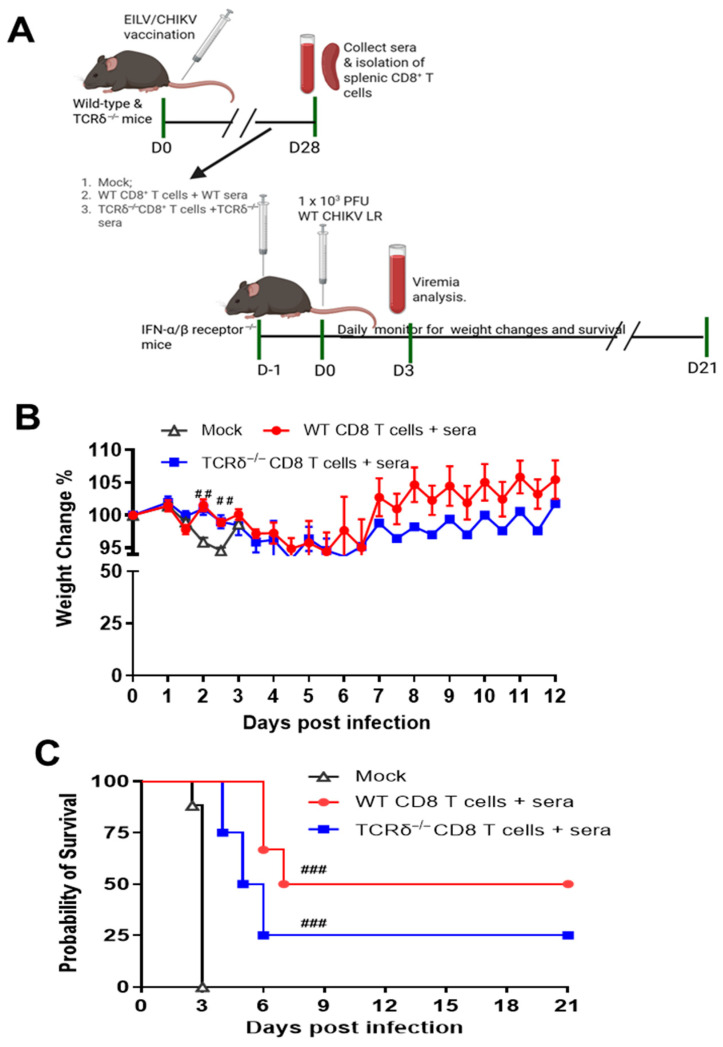
γδ T cell-mediated CD8^+^ T cells and antibodies are required for EILV/CHIKV-induced host protection against WT CHIKV infection. WT and TCRδ^−/−^ mice were vaccinated i.p. with 10^8^ PFU EILV/CHIKV. At 28 DPV, pooled sera and purified CD8^+^ T cells of EILV/CHIKV-vaccinated WT and TCRδ^−/−^ mice were transferred to AB6 mice 24 h before infection i.p. with 1000 PFU WT CHIKV LR strain. PBS-infected AB6 mice were used as the mock control. (**A**). Study design (Created in BioRender. Wang, T. (2025). https://BioRender.com/wcfie7o (accessed on 15 August 2025)). (**B**,**C**). Mice were monitored daily for weight changes and survival rate (n = 4 to 17). Weight changes are indicated by percentage using the weight on the day of infection as 100%. ### *p* < 0.001, or ## *p* < 0.01 compared to mock group.

CHIKV-specific antibody and B cell response are important for viral clearance and protect the host from persistent CHIKV infection [35,36]. Here, we collected sera from WT and TCRδ^−/−^ mice 28 DPV. TCRδ^−/−^ mice exhibited a reduced EILV/CHIKV-specific IgG response compared to the vaccinated WT mice (Figure 3A). In addition, TCRδ^−/−^ mice had significantly lower CHIKV E2-specific IgG1 and IgG2c responses and reduced neutralization antibody titers against WT CHIKV compared to the WT mouse group (Figure 3B–D). Next, we utilized conventional B-cell ELISpot to measure CHIKV-specific memory B cell (MBC)s in splenocytes of vaccinated mice. Because circulating MBCs do not actively secrete antibodies, we stimulated the splenocytes with the TLR7/8 agonist, R848, and rIL-2 in vitro for 5 days to convert MBCs into antibody secreting cells (ASC)s. At day 4, the frequency of MBCs specific for EILV/CHIKV in the vaccinated TCRδ^−/−^ mice was 4-fold lower than the vaccinated WT mice (Figure S3). CHIKV structural protein peptide pools were also used as antigens to detect CHIKV-specific MBCs (Figure 3E). At day 71, the frequency of MBCs specific for CHIKV capsid, E3, and E1 proteins but not E2 was significantly lower in EILV/CHIKV-vaccinated TCRδ^−/−^ mice than in the WT group. To determine the protective effects of EILV/CHIKV-induced humoral immunity, we next performed passive immunization in AB6 mice with pooled sera from EILV/CHIKV-vaccinated WT and TCRδ^−/−^ mice 28 DPV followed by infection with a lethal dose of WT CHIKV LR strain. There was significantly higher viremia at day 3 and more severe weight loss at days 4 and 5 in the TCRδ^−/−^ group compared to the WT group (Figure 3F,G). Both WT and TCRδ^−/−^ groups showed delayed mortality rates compared to the mock group, and 10% of the WT group survived of CHIKV infection. Compared to the WT group, TCRδ^−/−^ groups succumbed to infection significantly faster (Figure 3H). Furthermore, to determine if the addition of CD8^+^ T cells from the vaccinated mice increases host protection, we performed adoptive transfer into AB6 mice of both pooled sera and purified CD8^+^ T cells collected at day 28 DPV from EILV/CHIKV-vaccinated WT and TCRδ^−/−^ mice followed by infection with a lethal dose of WT CHIKV (Figure 4A). Both vaccinated WT and TCRδ^−/−^ groups showed a reduced weight loss at the early stage of infection (days 2 and 3) and increased survival rates compared to the mock group. The WT group showed a 50% survival from WT CHIKV infection, whereas the TCRδ^−/−^ group had a 25% survival rate (Figure 4B). Overall, these results suggest that γδ T cell-mediated CD8^+^ T cell and humoral immune responses following EILV/CHIKV vaccination are both required for the protection of the host from WT CHIKV infection.

### 3.4. γδ. T Cell Expanded in Response to EILV/CHIKV Vaccination, Induced Innate Cytokine Production and Promoted Antigen Presenting Cell (APC) Maturation

**Figure 5:**
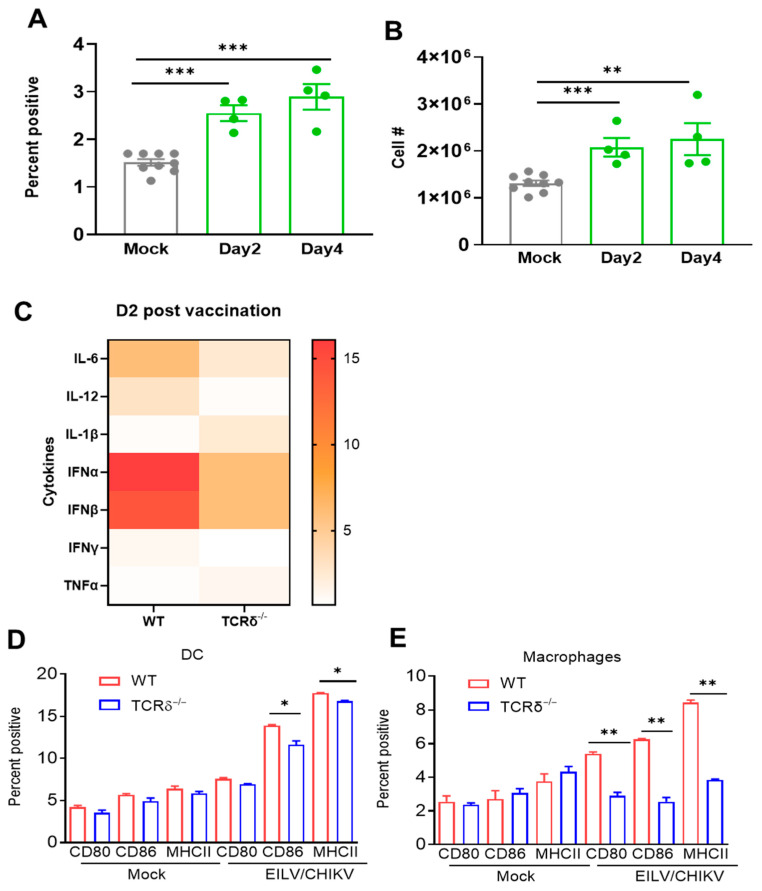
γδ T cells expanded quickly and promoted the induction of innate cytokine production and APC maturation following EILV/CHIKV vaccination. WT B6 mice were vaccinated i.p. with 10^8^ PFU EILV/CHIKV, or PBS (mock). Splenocytes were collected at 2 and 4 DPV, counted and stained for CD3 and TCR γδ and analyzed using flow cytometry. (**A**,**B**). Percent positive and total cell number of γδ T cells. n = 4–8. (**C**). At 2 DPV, blood cytokine levels were measured by Q-PCR assay. Data are presented as fold increase compared to the mock-vaccinated mice. n = 6. (**D**,**E**). At 5 DPV, splenic DCs and macrophages were stained for surface activation markers and analyzed by flow cytometry. *** *p* < 0.001, ** *p* < 0.01, or * p < 0.05 compared to mock or WT group.

To study the γδ T cell response to EILV/CHIKV vaccination, we vaccinated 6-week-old C57BL/6 mice with 10^8^ PFU of EILV/CHIKV or PBS (mock). At 2 and 4 DPV, splenocytes of EILV/CHIKV-vaccinated mice were isolated, counted and stained for phenotype. EILV/CHIKV vaccination induced 1.6-to-1.9-fold higher increases on both percentage and cell number of splenic γδ T cells at days 2 and 4 (Figure 5A,B). To further explore γδ T cell-mediated protective immunity, we measured innate cytokines in WT and TCRδ^−/−^ mice 2 DPV, including both type I IFNs, and proinflammatory cytokines in the blood.

TCRδ^−/−^ mice displayed lower levels of IFNα, IFNβ, IL-6, IL-12, and IFN-γ, but higher levels of IL-1β and TNF-α (Figure 5C). Dendritic cells (DCs) and macrophages are potent APCs during viral infection. At 5 DPV, splenocytes from vaccinated mice were stained with antibodies for DC (CD11c) and macrophage (F4/80) markers together with cell maturation and activation molecules, including CD80, CD86, and MHC class II. Compared to the mock group, the levels of CD80, CD86, and MHCII expression on DCs and macrophages of WT and TCRδ^−/−^ mice were significantly increased (Figure 5D,E**)**. Importantly, we noted that EILV/CHIKV-vaccinated TCRδ^−/−^ mice had significantly lower levels of CD80, CD86 and MHCII expression on DCs and macrophages than WT group. Overall, these results suggest that γδ T cells expanded following EILV/CHIKV vaccination and promoted APC activation and maturation, which further promoted the induction of protective adaptive immunity.

## 4. Discussion

EILV/CHIKV vaccination provides rapid and long-lasting protection against WT CHIKV infection in mice and non-human primates [21,22]. The underlying immune mechanisms of EILV/CHIKV-induced host protection are not clearly understood. γδ T cells are known to respond quickly and play a key role in the front line of defense of many microbial infections [37,38]. Here, we found that mock-vaccinated WT and TCRδ^−/−^ mice had similar levels of viremia following WT CHIKV infection. EILV/CHIKV-vaccinated TCRδ^−/−^ mice had increased weight loss and viremia levels upon WT CHIKV challenge. Moreover, EILV/CHIKV-vaccinated TCRδ^−/−^ mice had impaired CHIKV-specific CD8^+^ T cell and humoral immune responses. Together, our results suggest γδ T cells are not directly involved in controlling CHIKV infection. γδ T cells contribute to EILV/CHIKV-induced protective immunity by promoting T cell priming and antibody production to control CHIKV infection. Furthermore, both γδ T cell-mediated CD8^+^ T cells and antibody responses are required for EILV/CHIKV-induced host protection. Compared to EILV/CHIKV-induced CD8^+^ T cell responses, the vaccine-induced antibody responses appear to provide stronger protection.

We previously found that EILV/CHIKV induces APC activation upon in vivo vaccination, but not upon in vitro exposure to EILV/CHIKV, which suggests other host factors are involved in promoting APC activation and maturation in mice [24]. Here, we found TCRδ^−/−^ mice had reduced type I IFNs and proinflammatory cytokine production in sera at 2 DPV. Furthermore, TCRδ^−/−^ mice showed an impaired activation of DCs and macrophages at 5 DPV. γδ T cells can form a unique link between innate and adaptive immune responses [39,40,41,42]. Together, our results suggest that γδ T cells induce early innate cytokine production and promotes APC activation and maturation upon EILV/CHIKV vaccination.

CD8^+^ T cells are known to contribute to viral clearance and protecting the host from virus-induced inflammation during WT CHIKV infection [32,33]. Here, we noted that EILV/CHIKV-induced CD8^+^ T cells alone were not sufficient to protect mice from the mortality induced by a lethal dose (LD_100_, 80 PFU) of WT CHIKV LR strain infection, though these cells mitigated the weight loss at early time points of infection. One limitation of the adoptive transfer study is that the LD_100_ dose of the WT CHIKV infection may be too high to differentiate the protective effects of CD8^+^ T cells from the vaccinated WT and TCRδ^−/−^ mice. Nevertheless, following infection with a higher dose (12.5 LD_100_, 1000 PFU) of WT CHIKV infection, mice transferred with the immune sera of EILV/CHIKV-vaccinated WT and TCRδ^−/−^ mice were shown to have significantly delayed mortality rates. Furthermore, the adoptive transfer of both CD8^+^ T cells and immune sera of the vaccinated WT and TCRδ^−/−^ mice increased the survival rates to 50% and 25%, respectively. Thus, despite the dose limitation, these studies demonstrate the differential protective efficacy between the EILV/CHIKV-induced immune sera and CD8^+^ T cells responses. More importantly, these studies confirm that both EILV/CHIKV-induced humoral and cellular immune responses, which are mediated by γδ T cells, are required for protecting the host from WT CHIKV infection.

The EILV platform has been utilized efficiently in the development of candidate vaccines for several alphaviruses, including CHIKV, eastern (EEEV) and Venezuelan equine encephalitis viruses (VEEV) [21,43], and is known to induce quicker and more durable adaptive immunity than other vaccine platforms. Our results here suggest that γδ T cells expand in response to EILV/CHIKV vaccination and play an important role in mediating EILV/CHIKV-induced adaptive immunity. Identifying host factors involved in EILV/CHIKV induction will help further optimization and development of the vector-based CHIKV vaccines. Results from this study provide novel insights into the immune induction triggered by the EILV-based chimeric vaccines and the innate signaling pathways involved in γδ T cell activation.

## Data Availability

The data that support the findings of this study are available from the corresponding author upon reasonable request.

## Supplementary Materials

The following supporting information can be downloaded at: https://www.mdpi.com/article/10.3390/pathogens14090863/s1, Figure S1: Weight loss in EILV/CHIKV- vaccinated mice following WT CHIKV infection. Figure S2: T cell responses in WT and TCRδ^−/−^ mice following EILV/CHIKV vaccination. Figure S3. Memory B cell responses in WT and TCRδ^−/−^ mice following EILV/CHIKV vaccination.
