# Supplementary material for: γδ T Cells Mediate Protective Immunity Following Vaccination with an Insect-Based Chikungunya Fever Vaccine in Mice
Source: Pathogens. 2025 Aug 30;14(9):863. doi: 10.3390/pathogens14090863 (PMC12472593; doi:10.3390/pathogens14090863)
Supplement: Supplementary file 1 [file pathogens-14-00863-s001.zip › pathogens-3731327-supplementary.pdf]

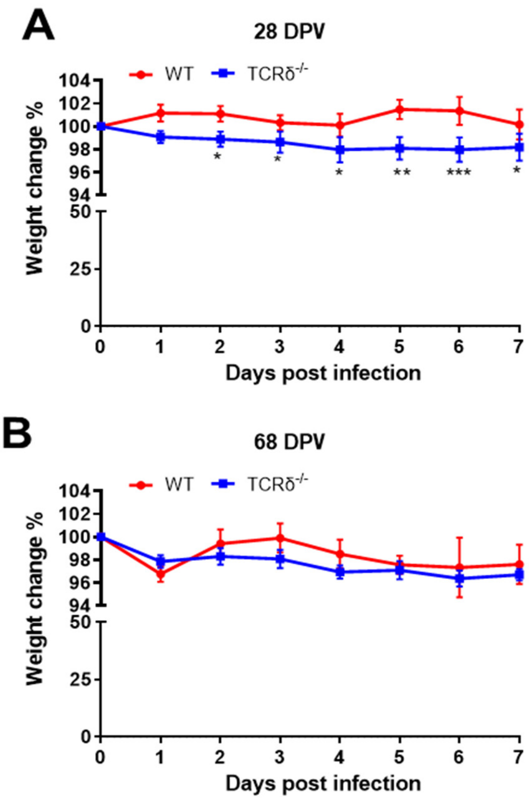

Figure S1: Weight loss in EILV/CHIKV- vaccinated mice following WT CHIKV infection. WT and TCRδ$^{-/-}$ mice were vaccinated with 10^8 PFU EILV/CHIKV or PBS (mock). **A-B.** At 28 (**A**) and 68 (**B**) DPV, mice were challenged with 5 x 10^5 PFU of CHIKV LR strain. Mice were monitored daily for morbidity and weight changes. Weight loss is indicated by percentage using the weight on the day of infection as 100%. *** *P* <0.001, ** *P* <0.01, or **P* < *0.05* compared to WT group.

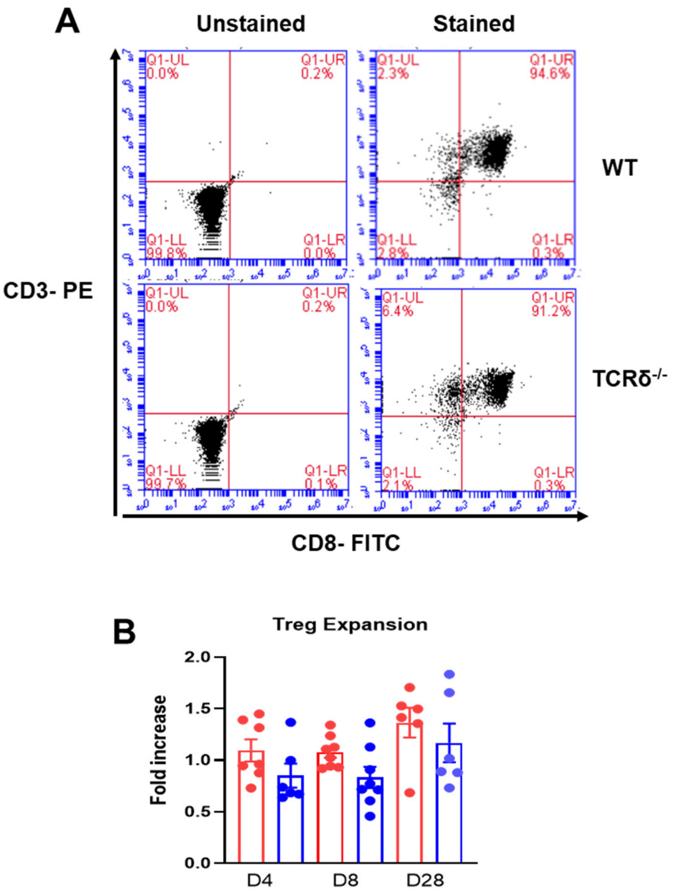

Figure S2. T cell responses in WT and TCRδ$^{-/-}$ mice following EILV/CHIKV vaccination. WT and TCRδ$^{-/-}$ mice were vaccinated with 10^8 PFU EILV/CHIKV, or PBS (mock). At 28 DPV, CD8$^+$ T cells were prepared from spleens of EILV/CHIKV-vaccinated WT or TCRδ$^{-/-}$ mice by negative selection using magnetic beads. The purity of CD8$^+$ T cells was checked by staining cells with antibodies for CD3 and CD8 and analyzed by flow cytometry. **A.** Representative dot-plots of CD8$^+$ T cell staining of each group were shown. **B.** At different DPVs, splenocytes were stained for Treg markers. Data represents fold increase compared to the mock-vaccinated groups.

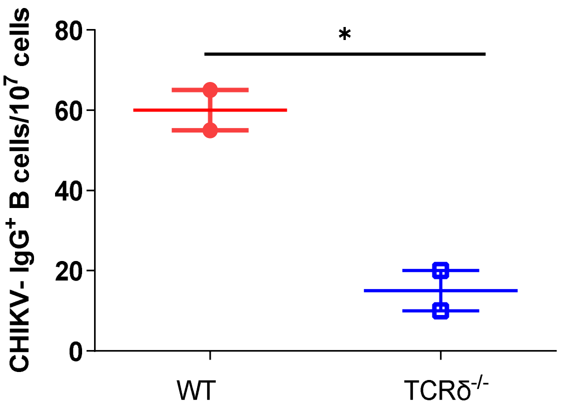

Figure S3. Memory B cell responses in WT and TCRδ$^{-/-}$ mice following EILV/CHIKV vaccination. WT and TCRδ$^{-/-}$ mice were vaccinated i.p. with 10^8 PFU EILV/CHIKV. At 4 DPV, splenocytes were isolated and CHIKV- specific MBC responses were determined by ELISPOT analysis using EILV/CHIKV as an antigen. Frequencies of CHIKV antibody secreting cells per 10^7 cells were shown. n = 2. *$P < 0.05$ compared to WT group.
